# Phytochemical diversity and antioxidant capacity of citrus cultivars in Zhejiang: a phenolic profiling study for resource evaluation and differentiation

**DOI:** 10.3389/fnut.2026.1799170

**Published:** 2026-04-01

**Authors:** Weiqing Zhang, Yi Li, Wei Li, Xianju Feng, Mingxia Wen, Tianyu Wang, Bei Huang, Mei Lin

**Affiliations:** Institute of Citrus Research, Zhejiang Academy of Agricultural Sciences, Taizhou, China

**Keywords:** antioxidant activity, flavonoids, phenolic acids, PCA, HCA

## Abstract

To elucidate the under-characterized phytochemical diversity of regionally important citrus germplasm, this study performed a comparative analysis of phenolic profiles and antioxidant activities in seven cultivars from Zhejiang Province, including commercial, locally adapted and emerging varieties. Using UPLC-MS/MS, 17 flavonoids and 11 phenolic acids were quantified in peel and pulp tissues, revealing significant metabolite differentiation among varieties. The hybrid variety Gaocheng (pomelo × orange) displayed a distinct intermediate chemotype. It was characterized by the concurrent accumulation of naringin, and hesperidin. Notably, the peels of Ponkan, Bendizao, Gaocheng, and Miyagawa wase were identified as rich sources of bioactive PMFs. All peel extracts exhibited strong antioxidant activity, with Gaocheng showing the highest comprehensive antioxidant capacity. PCA and HCA revealed that phenolic composition was strongly associated with genetic background, classifying the varieties into three distinct groups. Collectively, these findings provide a foundation for the utilization of citrus by-products, cultivar authentication, and breeding strategies for enhanced bioactivity.

## 1 Introduction

Citrus is China’s largest fruit crop, ranking first in the world for both planted area and production (1). The country’s rich germplasm encompasses major types, including mandarins, sweet oranges, pomelos, lemons, kumquats, and hybrids (2). Citrus fruits are valuable sources of metabolites including organic acids, sugars, and vitamins, as well as bioactive compounds such as alkaloids and polyphenols (3–5). Notably polyphenols, which are strongly associated with health benefits due to their antioxidant (6–8), anti-carcinogenic (9), anti-inflammatory (10), and other bioactive properties. The accumulation of these compounds is primarily governed by genetic factors, especially cultivar identity, and further influenced by environmental conditions such as climate, soil, and fruit developmental stage.

Technological advances have enabled a growing number of metabolomic studies aimed at uncovering discrepancies in citrus quality. Liang et al. provided valuable genomic and metabolomic insights into bioactive compound variations in citrus peels across 299 citrus accessions and evaluated antioxidant and anticancer capacities using 219 varieties (11). In a more recent study, Peng et al. characterized the primary metabolic networks underlying citrus flavor diversification, with phenolic acids providing secondary modulation (12). These studies have established the broad metabolic landscape of citrus at the population level. However, an important knowledge gap remains regarding comprehensive, comparative analyses of pulp metabolites in unique, locally adapted, and emerging cultivars from specific, major production regions. Our previous work analyzed differences in secondary metabolites among 11 citrus varieties from Zhejiang Province (13). Nevertheless, it did not comprehensively assess the corresponding antioxidant activities, leaving the functional implications unexplored. Filling these gaps is essential for a complete understanding of citrus phytochemical diversity and functional potential, particularly for guiding the targeted utilization of regionally important germplasm.

To address these gaps, seven citrus cultivars were strategically selected to represent distinct genetic, adaptive and commercial backgrounds. (i) A dominant commercial variety, Miyagawa wase (*Citrus unshiu* Mac. Miyagawa wase), was included to uncover its latent nutritional value, thereby potentially enhancing its competitiveness in health-conscious markets. (ii) Traditional locally adapted cultivars were also incorporated, including Bendizao (*Citrus succosa* Hort. Ex Tanaka), Gaocheng (*C. grandis* × *C. sinensis*), Ponkan (*Citrus reticulata* Blanco), and Yuhuanyou (*Citrus grandis* Osbeck). Having been cultivated for extended periods under distinct regional agro-climatic conditions, these varieties may possess unique phenolic profiles shaped by long-term environmental adaptation. (iii) Emerging commercial varieties, Hongmeiren (*Citrus hybird* Hongmeiren) and Cocktail grapefruit (*Citrus paradisi* Cocktail) are gaining substantial market interest due to their sensory or perceived nutritional qualities. However, their bioactive compositions and antioxidant potential remain scientifically underexplored. This deliberate selection strategy enables an expansion of the phytochemical diversity within citrus.

To evaluate bioactivity, three assays (DPPH, ABTS, and FRAP) were employed to assess the antioxidant activities of the samples. The antioxidant potency composite (APC) index was subsequently calculated for overall ranking. This approach enabled the identification of peel extracts with superior antioxidant potential among the tested cultivars.

The practical relevance of this work is further underscored by the need to valorize citrus processing by-products. About one-third of citrus production is processed, generating large quantities of peel waste, which may cause environmental pollution if not used (14). Citrus peel is a concentrated source of polyphenols and is known for its high antioxidant activity (15). This property positions it as a high-potential raw material for nutraceutical, cosmetic, and food industries (16). However, achieving efficient and targeted valorization of peel waste depends on a precise understanding of its chemical composition and antioxidant capacity, which are known to vary considerably among different varieties. Therefore, cultivar-specific phytochemical profiling is a critical prerequisite for the developing utilization pathways.

Based on these considerations, the present study was designed to: (i) systematically compare the phenolic composition and antioxidant capacity of seven regionally important citrus varieties representing commercial, locally adapted, and emerging categories; (ii) elucidate tissue-specific distribution patterns of phenolics; and (iii) identify cultivars with superior antioxidant potential for targeted applications. By integrating expanded cultivar coverage and antioxidant characterization, this work is expected to provide foundational data for citrus breeding, biosynthetic studies, and citrus processing by-products utilization.

## 2 Materials and methods

### 2.1 Chemicals and materials

Seven citrus varieties were collected from the Zhejiang Citrus Research Institute Germplasm Nursery (Zhejiang Province, China). Fruits were harvested at commercial maturity stage. For each cultivar, three trees with similar age and vigorous growth were selected. Twelve healthy fruits of similar size were randomly picked from each tree, resulting in a total of 36 fruits per cultivar. These were pooled and randomly divided into three groups of 12 fruits each, representing three independent biological replicates. Fruits were separated into peel and pulp, with both tissues being derived from the same individual fruits within each replicate. All samples were oven dried at 40 °C, ground into powder, and stored at −80 °C for subsequent analysis.

HPLC-grade methanol was purchased from Merck (Darmstadt, Germany). Formic acid was bought from TEDIA (Fairfield, USA). Flavonoid and phenolic acid standards were purchased from Sigma-Aldrich (St. Louis, Missouri, USA). All the other analytical-grade reagents were obtained from Sinopharm Chemical Reagent Co., Ltd. (Shanghai, China). Ultra-pure water was prepared using the Milli-Q water purification system (Millipore Corporation, Billerica, USA).

### 2.2 Extraction of flavonoids

Flavonoids extraction was carried out according to a previous study with some modifications (17). Briefly, citrus tissues were ultrasonically extracted with 10 mL of 80% ethanol for 30 min using an ultrasonic bath (SK250HP, Shanghai Kedao Ultrasonic Instruments Co., Ltd., Shanghai, China) and centrifuged at 10,000 × *g* for 10 min at 4 °C (Sorvall ST 16R, Thermo Fisher Scientific, Germany). Each extraction procedure was repeated three times. All the supernatants were combined and adjusted to a final volume of 30 mL with 80% ethanol. The extracts obtained were filtered through a 0.45 μm membrane filter for UPLC-MS/MS analyses.

### 2.3 Extraction of phenolic acids

The extraction process was performed according to a prior study with some modifications (13). Powdered citrus tissues were extracted with NaOH solution (4 mol/L) by shaking for 2 h. The pH of extraction solution was adjusted to 2.0 using HCl solution (6 mol/L), followed by centrifugation at 10,000 rpm for 10 min. The supernatant was collected, purified using a solid-phase extraction column, and eluted with methanol. The eluate was finally diluted to a volume of 5 mL with methanol. The extracts were filtered through a 0.45 μm membrane filter and used for the following analysis.

### 2.4 UPLC-MS/MS analysis of flavonoids

Flavonoid extracts were analyzed using a UPLC-MS/MS system (AB SCIEX, USA). Separation was performed on a Kinetex C18 column (2.6 μm, 3.0 × 100 mm) maintained at 40 °C. A 2.0 μL sample was injected and the mobile phase was consisted of 5 mM ammonium acetate (solution A) and methanol (solution B) with a gradient elution as follows: a linear gradient from 50% B to 80% B in 4 min, followed by an isocratic step of 80% B for 2 min, and a back to 50% B in 0.5 min. The flow rate was 0.3 mL/min. All experiments were conducted in triplicate, and results were expressed as mg/kg dry weight (DW).

### 2.5 UPLC-MS/MS analysis of phenolic acids

A UPLC-MS/MS system and a Kinetex C18 (2.6 μm, 3.0 mm × 100 mm) was used to separate and analyze the phenolic acids in citrus extracts. A 2.0 μL sample was injected and the mobile phase consisted of 1% formic acid (solution A) and methanol (solution B) and delivered isocratically at a ratio of 50:50 (A:B). The column temperature was at 40 °C and the flow rate was 0.3 mL/min. All experiments were conducted in triplicate, and results were expressed as mg/kg dry weight (DW).

### 2.6 Measurement of antioxidant activity

The antioxidant capacities of citrus peel extracts were evaluated using DPPH, ABTS, and FRAP assay kits (Boxbio, Beijing, China). ABTS and DPPH values were expressed as μmol Trolox equivalents per gram of dry weight (μmol TE/g DW). The FRAP value of samples was expressed as μmol Fe^2+^ equivalents per gram of dry weight (μmol Fe^2+^/g DW).

### 2.7 Statistical analysis

All assays were conducted on three independent biological replicates per cultivar. Results were expressed as mean ± standard deviation (SD). One-way analysis of variance (ANOVA) was performed to assess overall differences among cultivars, followed by the Least Significant Difference (LSD) post-hoc test for multiple comparisons. Differences were considered statistically significant at *p* < 0.05. Origin 2025b was used for graphical presentation (Origin Lab Corporation, USA). Heatmaps, PCA and HCA were performed using R packages.

## 3 Results

### 3.1 Qualitative and quantitative analysis of components in citrus peel

**Figure 1:**
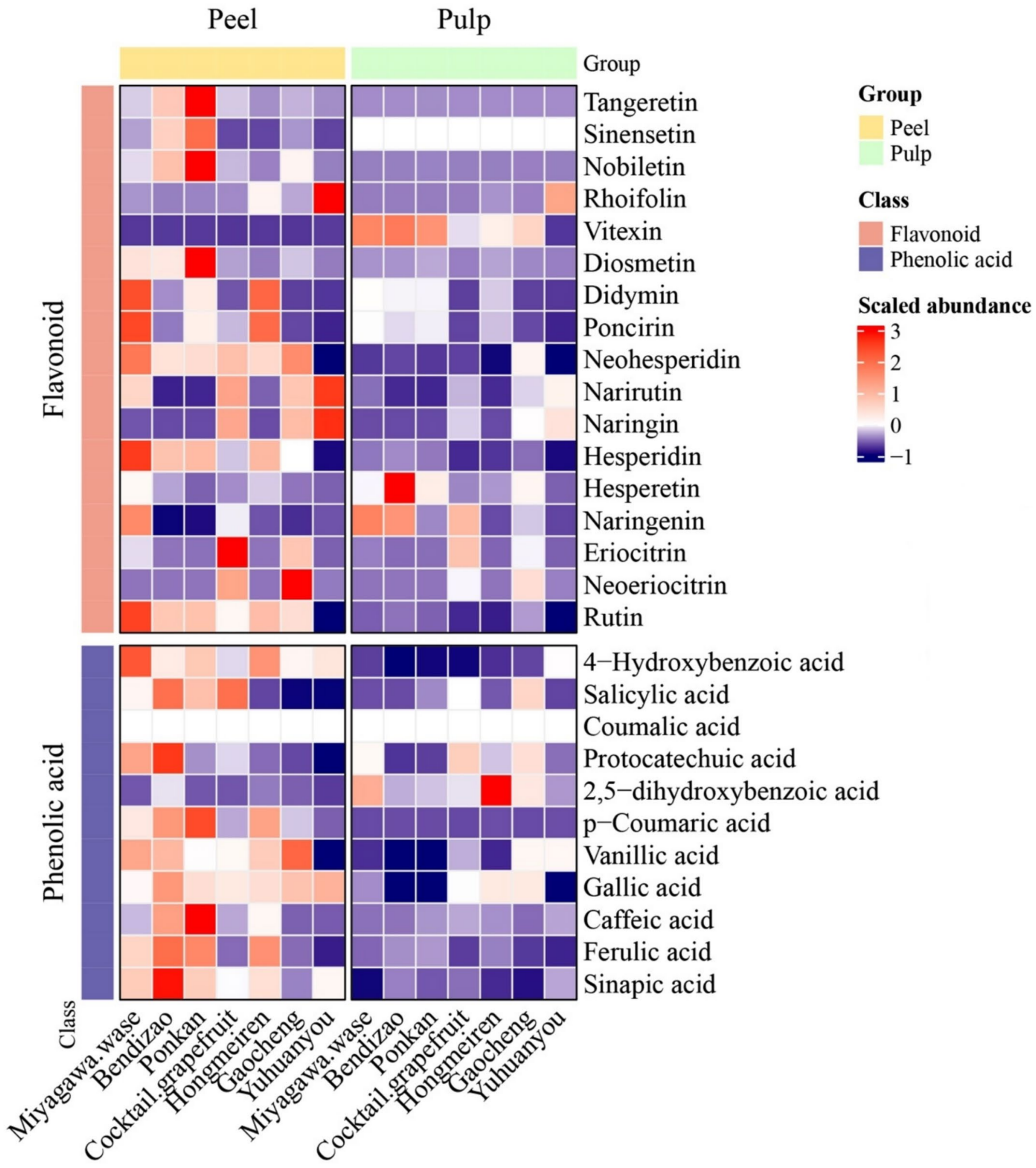
Heatmap of phenolic compounds in pulp and peel of seven citrus varieties. The data is normalized, with red representing a higher relative content and blue representing a lower relative content.

Citrus peel, a major by-product of citrus processing, was confirmed to be rich in bioactive compounds. The polyphenolic profiles of the studied citrus varieties are shown in Figure 1 (Supplementary Table S1 and S3). Flavonoids varied among varieties, ranging from 51,172.56 to 104,256.17 mg/kg. Miyagawa wase contained the highest flavonoid content, approximately 2.0 times that of Bendizao (the lowest). Flavanones were the most abundant flavonoids subclass, ranging from 30,388.21 to 71,035.36 mg/kg. Hesperidin was the major flavonoid in Miyagawa wase, Bendizao, Ponkan, and Hongmeiren, with the highest concentration observed in Miyagawa wase. In contrast, naringin was the most abundant flavonoid in Cocktail grapefruit, Yuhuanyou, and Gaocheng. Neohesperidin was highest in Miyagawa wase and lowest in Yuhuanyou. Notably, neoeriocitrin was detected only in Gaocheng (6,535.24 mg/kg), Cocktail grapefruit (3,278.16 mg/kg), and Yuhuanyou (40.56 mg/kg). The highest content of flavones was in Yuhuanyou, much higher than other varieties. Polymethoxyflavones (PMFs) were measured, with Ponkan containing the highest contents of nobiletin (8,316.06 mg/kg), tangeretin (3,714.90 mg/kg) and sinensetin (432.11 mg/kg). Regarding flavonols, the rutin content was highest in Miyagawa wase (32,170.54 mg/kg) and lowest in Cocktail grapefruit (4.31 mg/kg).

Ten phenolic acids were detected in the peels, with total contents ranging from 161.60 to 3,933.01 mg/kg (Figure 1; Supplementary Table S2–S3). The lowest phenolic acid content was found in Yuhuanyou, while Bendizao had the highest content, approximately 24.3 times that of the former. Ferulic acid was the dominant phenolic acid in all varieties except Yuhuanyou, where sinapic acid predominated. Notably, Bendizao had the highest contents of both ferulic acid and sinapic acid, which were significantly greater than those in other samples. The highest levels of caffeic acid and p-coumaric acid were found in Ponkan. In addition, low levels of 2,5-dihydroxybenzoic acid and gallic acid were present in the peel. Overall, phenolic acids were abundant in Bendizao, Ponkan, Hongmeiren, and Miyagawa wase, but were present at relatively low levels in Yuhuanyou.

### 3.2 Qualitative and quantitative analysis of components in citrus pulp

Citrus pulp, as the directly edible portion, is rich in bioactive compounds. Flavonoid content and composition in the pulp varied across varieties (Figure 1; Supplementary Table S4). Total flavonoid contents ranged from 8,461.17 (Hongmeiren) to 36,300.60 mg/kg (Gaocheng). Flavanones were the predominant class overall. The identity of the secondary major component differed: flavonols in Cocktail grapefruit and Gaocheng, and flavones in other varieties. Bendizao had the highest flavone content (5,526.33 mg/kg), about 7.8 times that of Yuhuanyou (704.82 mg/kg). Similar to citrus peels, naringin was the main flavonoid in the pulp of Cocktail grapefruit, Gaocheng, and Yuhuanyou, and was highest in Yuhuanyou (13,705.90 mg/kg). In contrast, hesperidin was the main flavonoid compound in the remaining four varieties. Flavonol contents ranged from 4.31 to 6,343.83 mg/kg, being highest in Gaocheng and lowest in Yuhuanyou. The contents of PMFs in the pulp were much lower than those in the peel. Notably, rhoifolin was detected only in Yuhuanyou (699.15 mg/kg), Hongmeiren (31.33 mg/kg), and Gaocheng (6.72 mg/kg). Eriocitrin and neoeriocitrin were present at significantly higher levels in Cocktail grapefruit and Gaocheng compared to other varieties. Eriocitrin was undetectable in Yuhuanyou, and neoeriocitrin was absent in Miyagawa wase, Ponkan, and Hongmeiren.

The total phenolic acid content in pulp was much lower than that in peel for all varieties except Yuhuanyou, ranging from 215.74 to 647.23 mg/kg (Figure 1; Supplementary Table S4). Ponkan, Bendizao, Hongmeiren, and Miyagawa Wase were rich in phenolic acids, while Yuhuanyou and Gaocheng were relatively poor. Similar to the pattern in peels, ferulic acid was found to be the most abundant phenolic acid in most varieties, except in Yuhuanyou where sinapic acid was the main component. The ferulic acid content varied across varieties, with the highest concentration in Ponkan (539.54 mg/kg) and the lowest in Yuhuanyou (52.35 mg/kg). Additionally, the highest contents of caffeic acid (45.79 mg/kg), vanillic acid (40.40 mg/kg) and 4-hydroxybenzoic acid (14.18 mg/kg) were found in Cocktail grapefruit, Gaocheng and Yuhuanyou, respectively.

### 3.3 Comparison of phenolic components in the peel and pulp

The accumulation patterns of phenolic compounds exhibited significant variations among citrus varieties and between tissues (Figure 1). A consistent trend observed across all cultivars was the substantially greater abundance of total flavonoids compared to phenolic acids. Furthermore, concentrations of most individual phenolic compounds were higher in the peel than in the pulp. However, the rank order of varieties based on total flavonoid content differed markedly between tissues. In the peel, Miyagawa wase contained the highest total flavonoid level, followed by Gaocheng, with Bendizao having the lowest. In contrast, in the pulp, Gaocheng accumulated the highest flavonoid content, followed by Bendizao, while Hongmeiren had the lowest. Similarly, phenolic acid content varied considerably among varieties and tissues. In the peel, Bendizao had the highest phenolic acid content and Yuhuanyou the lowest, whereas in the pulp, Ponkan showed the highest level and Yuhuanyou remained the lowest. Notable exceptions to the general pattern of peel-dominated accumulation were identified. The contents of vitexin and total flavones were significantly higher in the pulp than in the peel in all varieties except Yuhuanyou. Furthermore, in Yuhuanyou, the ferulic acid content in the pulp was approximately twice that in the peel.

### 3.4 Correlation analysis of phenolic compounds in citrus

**Figure 2:**
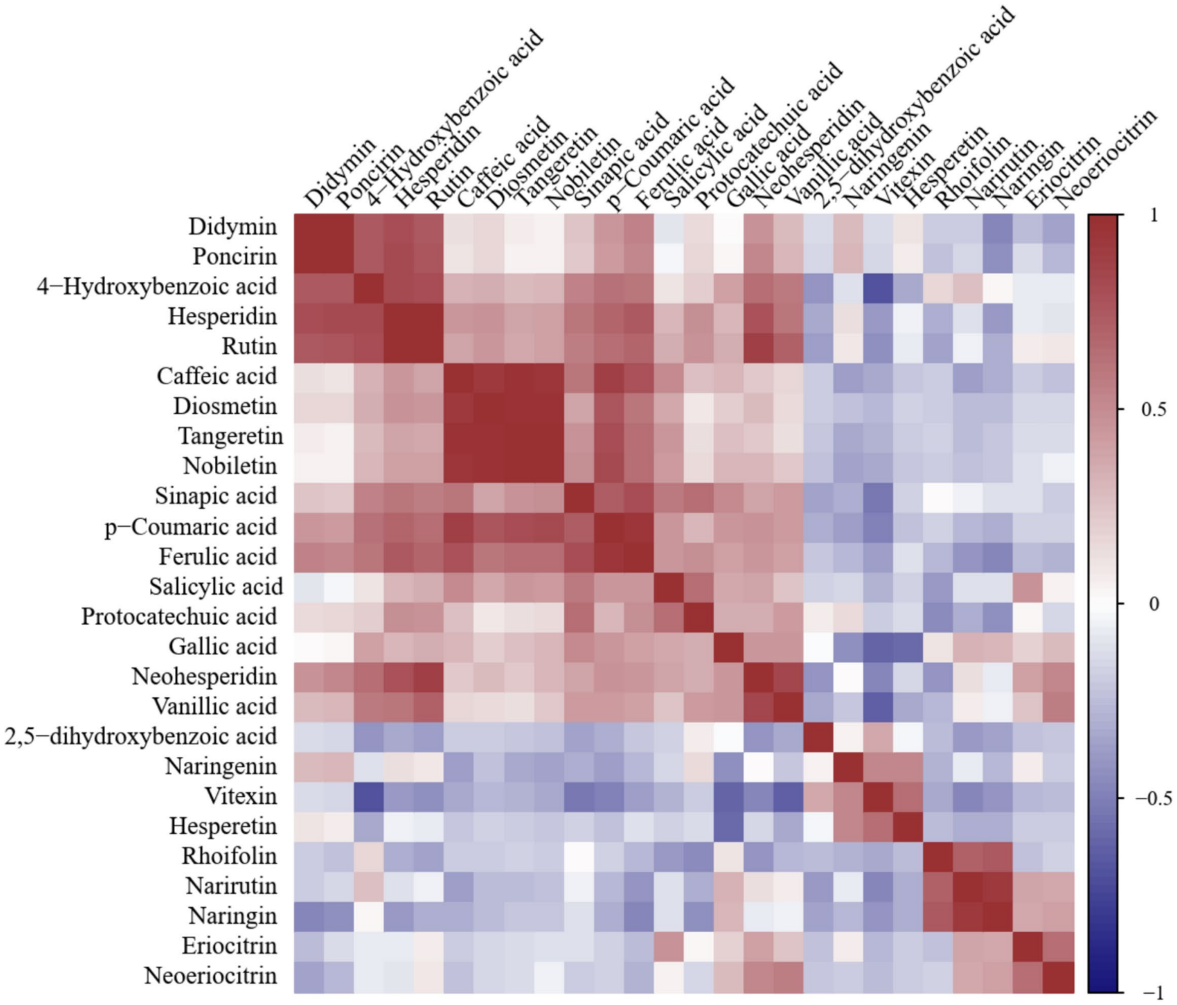
Correlation heatmap of phenolic compounds in citrus.

Pearson correlation analysis was performed to evaluate the relationships among phenolic compound contents in seven citrus varieties (Figure 2; Supplementary Table S5). The results indicated that PMFs, flavonols, and most phenolic acids (except 2,5-dihydroxybenzoic acid) showed positive correlations with each other, but most of them were negatively correlated with flavanones. Significant positive correlations were observed among tangeretin, nobiletin, diosmetin, p-coumaric acid and caffeic acid (*r* ≥ 0.779, *p* < 0.05). Similarly, rutin, didymin, poncirin, hesperidin, and 4-hydroxybenzoic acid had strong positive correlations with each other (*r* ≥ 0.744, *p* < 0.05), while 4-hydroxybenzoic acid was negatively correlated with vitexin (*p* < 0.05). Within the flavanones, hesperidin showed positive correlations with didymin, poncirin, neohesperidin, and naringenin, but was negatively associated with other flavanones. In contrast, 2,5-dihydroxybenzoic acid was positively correlated with vitexin, naringenin and protocatechuic acid, while it showed negative correlations with most other metabolites.

### 3.5 PCA and HCA analyses in citrus

**Figure 3:**
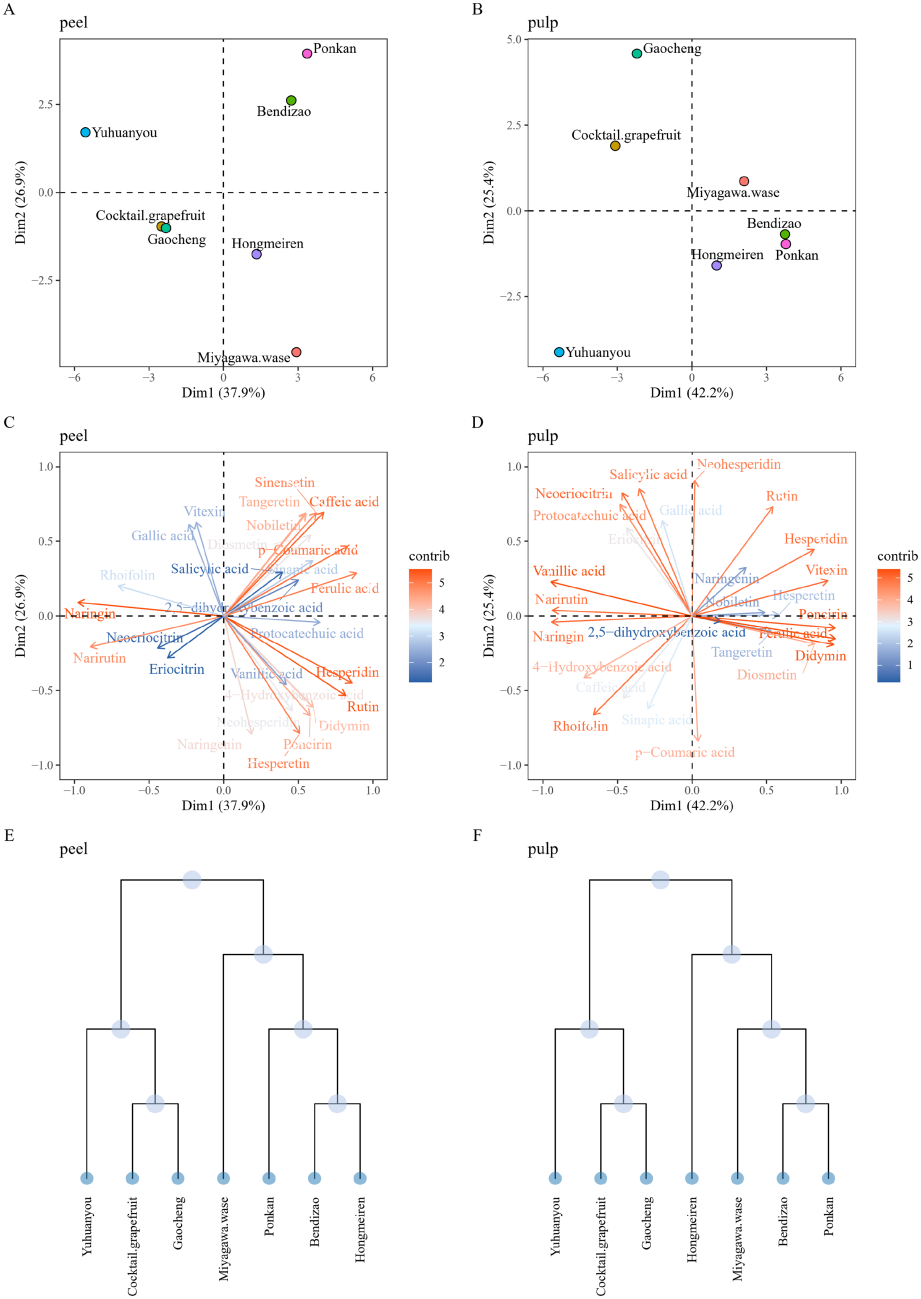
Score plots of principal components of seven citrus varieties based on phenolic profiles in the pulp and peel **(A, B)**. Loading plots of principal components from the PCA results based on metabolite data of the pulp and peel **(C, D)**. Hierarchical clustering of the pulp and peel **(E, F)**.

Principal component analysis (PCA) and hierarchical cluster analysis (HCA) were employed to classify the seven citrus varieties based on their phenolic profiles. PCA revealed distinct compositional patterns between peel and pulp tissues (Figure 3). The spatial distribution of samples in the score plot reflected the differences in the metabolite composition across the studied varieties. Based on the similarity of their metabolite profiles, the varieties were classified into four distinct groups. Notably, peel samples of Bendizao and Ponkan clustered in the first quadrant, a position correlated with higher loadings of PMFs, caffeic acid, ferulic acid, and p-coumaric acid. This spatial co-localization suggests that these two varieties accumulate relatively higher levels of these compounds in their peel tissues. Similarly, pulp samples of the same varieties grouped in the fourth quadrant, a cluster associated with elevated loadings of poncirin, ferulic acid, and didymin, indicating a relative enrichment of these metabolites in the pulp.

### 3.6 Antioxidant activity and correlation analysis in citrus

Antioxidant activities varied among the seven citrus peel samples (Table 1). The average DPPH value was 51.37 μmol/g. Gaocheng showed the highest value at 71.35 μmol/g, 2.5 times that of Yuhuanyou which had the lowest. ABTS values ranged from 65.13 to 89.30 μmol/g, with the lowest value observed in Yuhuanyou and the highest in Gaocheng. FRAP values varied significantly among varieties, in descending order as follows: 335.62 μmol/g (Miyagawa wase), 302.93 μmol/g (Bendizao), 290.43 μmol/g (Hongmeiren), 250.61 μmol/g (Gaocheng), 235.64 μmol/g (Ponkan), 206.86 μmol/g (Cocktail grapefruit), and 74.61 μmol/g (Yuhuanyou). Accordingly, Miyagawa wase exhibited the strongest FRAP-based antioxidant capacity, while Yuhuanyou had the weakest. Given that the three methods gave inconsistent antioxidant rankings across varieties, an overall APC index was calculated for each variety according to the method described by Seeram et al. (18). The APC index ranged from 43.87 to 88.87, indicating that Gaocheng, Miyagawa wase, and Ponkan peel extracts possessed relatively high overall antioxidant capacity among the tested samples.

**Table 1:** Antioxidant capacity and APC index in peels of seven citrus varieties.

Varieties	DPPH (μmol/g)	ABTS (μmol/g)	FRAP (μmol/g)	APC	Rank
Miyagawa wase	48.19 ± 1.44 d	75.25 ± 1.93 bc	335.62 ± 10.27 a	81.37 ± 1.00 b	2
Bendizao	49.02 ± 1.19 d	67.72 ± 3.04 de	302.93 ± 11.13 b	75.91 ± 2.64 d	5
Ponkan	62.84 ± 3.32 b	78.98 ± 2.25 b	235.64 ± 6.84 e	79.82 ± 1.10 bc	3
Cocktail grapefruit	47.17 ± 0.31 d	67.18 ± 3.00 e	206.86 ± 4.36 f	65.63 ± 1.04 e	6
Hongmeiren	51.98 ± 0.52 c	71.47 ± 1.56 cd	290.43 ± 3.96 c	77.41 ± 0.62 cd	4
Gaocheng	71.35 ± 1.56 a	89.30 ± 3.13 a	250.61 ± 5.77 d	88.87 ± 2.29 a	1
Yuhuanyou	29.05 ± 1.04 e	65.13 ± 0.15 e	74.61 ± 2.43 g	43.87 ± 0.69 f	7
Average	51.37 ± 12.70	73.57 ± 8.27	242.38 ± 81.63	73.27 ± 14.01	

Data are presented as mean ± standard deviation, *n* = 3. Different lowercase letters indicate significant differences between samples (*p* < 0.05). Different lowercase letters in the same column indicate significant difference at 0.05 level.

**Figure 4:**
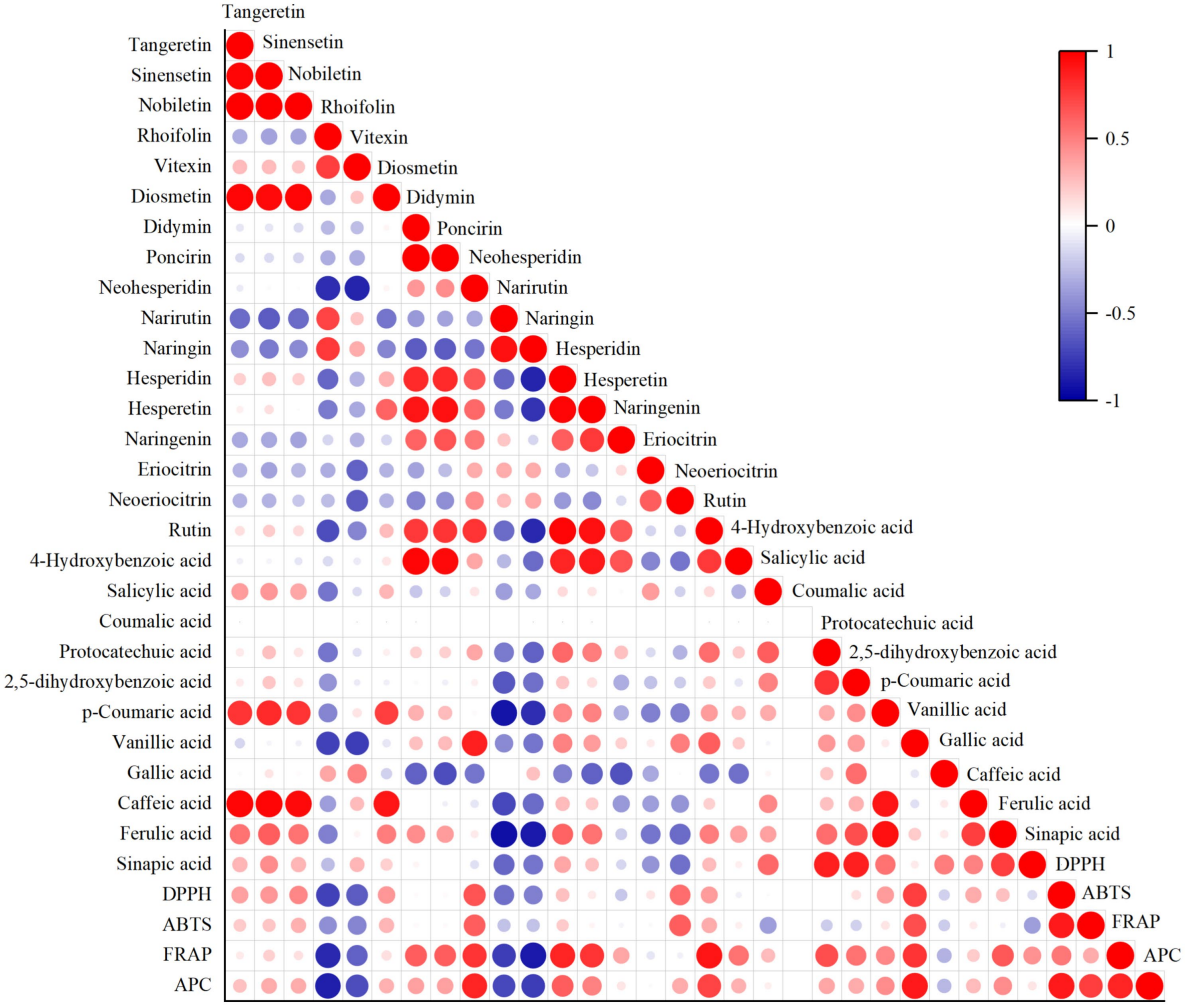
Pearson correlation analysis of the antioxidant activity and phenolic compound content in the peel of seven citrus cultivars. Positive correlations are shown in red, negative correlations in blue.

A Pearson’s correlation analysis was performed to determine the relationships between phenolic contents and antioxidant activities across the seven citrus varieties (Figure 4). The results revealed that DPPH, FRAP, and ABTS values were positively correlated with the contents of neohesperidin, PMFs, rutin, hesperidin, vanillic acid, and p-coumaric acid. Conversely, these antioxidant activity measures were negatively correlated with the concentrations of rhoifolin, vitexin, narirutin, naringin, and gallic acid.

## 4 Discussion

Flavonoids, a major class of polyphenolic secondary metabolites, are key determinants of the nutritional and bioactive quality of citrus fruits (19, 20). Consistent with the well-documented pattern, our study confirmed significant tissue-specific accumulation, with flavonoid concentrations markedly higher in the peel than in the pulp across all seven investigated cultivars (21, 22). This distribution likely arises from the biosynthetic localization of these compounds.

Importantly, pronounced varietal differences in flavonoid composition and abundance were observed. Flavanones, including hesperidin and naringin, constituted the dominant flavonoid class. Their relative abundance effectively delineated genetic groups. Cultivars with mandarin or sweet orange ancestry (Miyagawa Wase, Bendizao, Ponkan, Hongmeiren) exhibited high levels of hesperidin, a finding consistent with previous study (23). In contrast, varieties related to pomelo or grapefruit (Cocktail grapefruit, Yuhuanyou) accumulated naringin as their major flavonoid (24). This variation in flavanone accumulation aligns with the large-scale metabolomic profiling of 299 citrus accessions, which demonstrated that metabolic profiles are largely determined by genetic background (11). The hybrid cultivar Gaocheng (pomelo×orange) displayed a distinct intermediate chemotype. It was characterized by the concurrent accumulation of naringin, and hesperidin at relatively high levels. While previous targeted analyses have profiled primary metabolites and phenolic acids across five citrus species, the present study extends this work by quantifying a broader range of flavonoids (12). Moreover, building upon our previous work, which focused primarily on hybrid citrus varieties, we examined major commercial and locally adapted varieties, thereby enhancing practical relevance (13). By integrating uncharacterized local germplasm with the methodological distinction, this study provides a more comprehensive profile of metabolite diversity in regionally important germplasm. PMFs were detected predominantly in the peel, aligning with the previous study (25). Notably, the high accumulation of tangeretin and nobiletin in cultivars such as Ponkan and Bendizao identifies them as promising sources of these bioactive PMFs (26, 27).

Phenolic acids also exhibited distinct varietal and tissue-specific variation. Hydroxycinnamic acids, mainly ferulic acid, generally predominated over hydroxybenzoic acids (28). A notable exception was Yuhuanyou, whose pulp contained higher total phenolic acids than its peel, positioning it as a distinctive source of these bioactives. Discrepancies in the predominant phenolic acid types between our study and prior literature likely reflect differences in genetic background, cultivation conditions, and analytical methodologies (1).

DPPH, ABTS, and FRAP assays were used to evaluate the antioxidant activities of the seven citrus peel extracts. Variation was observed among the methods, likely reflecting their different reaction mechanisms. Therefore, the APC index was employed for overall ranking, revealing Gaocheng as the cultivar with the highest composite value. Furthermore, correlation analysis results showed that phenolic metabolite contents were associated with antioxidant capacity, consistent with previous studies (29, 30). However, as antioxidant capacity was assessed using crude extracts, the observed activities likely reflect the integrated effects of the complex phenolic mixture. The possibility of synergistic or antagonistic interactions cannot be excluded. Accordingly, these correlative findings should be regarded as hypothesis-generating, warranting further validation through isolation and reconstitution studies. Notably, the high APC values exhibited by local cultivars such as Gaocheng highlight the potential of their peels as rich sources of natural antioxidants for functional food or preservative applications.

Phenolic profiles could serve as phytochemical markers for elucidating genetic backgrounds and citrus classification. Citrus classification is complicated due to complex morphology and frequent hybridization. Multivariate analysis (PCA and HCA) based on phenolic composition grouped the seven varieties into three groups. The results indicated that closely related varieties exhibited similar metabolite profiles and converged into one group. This chemotaxonomic clustering provides metabolic validation of phylogenetic relationships and suggests that phenolic profiling could complement genotypic approaches for variety identification.

## 5 Conclusion

This study provides a comprehensive phytochemical characterization of both peel and pulp tissues from seven citrus cultivars, with a focus on regionally important germplasm. The peels of Ponkan, Bendizao, Gaocheng, and Miyagawa wase were identified as rich sources of bioactive PMFs, suggesting their potential for valorization as functional food ingredients or preservative agents. Notably, Gaocheng, a hybrid of pomelo and orange, displayed a distinct intermediate flavanone profile, characterized by the co-accumulation of naringin, and hesperidin. Its peel extracts also exhibited superior overall antioxidant capacity, indicating its potential as a candidate for further bioactivity-guided research. Multivariate analysis based on phenolic profiles clearly distinguished the cultivars. These findings provide a foundational dataset for breeding programs aimed at enhancing bioactive compound accumulation in citrus. Collectively, this work extends the understanding of phytochemical diversity in locally adapted germplasm and offers insights for the targeted utilization of citrus resources.

## Data Availability

The raw data supporting the conclusions of this article will be made available by the authors, without undue reservation.
