# Supplementary material for: Phytochemical diversity and antioxidant capacity of citrus cultivars in Zhejiang: a phenolic profiling study for resource evaluation and differentiation
Source: Front Nutr. 2026 Apr 1;13:1799170. doi: 10.3389/fnut.2026.1799170 (PMC13101761; doi:10.3389/fnut.2026.1799170)

**Phytochemical diversity and antioxidant capacity of citrus cultivars in Zhejiang: A phenolic profiling study for resource evaluation and differentiation**

**Weiqing Zhang*, Yi Li, Wei Li, Xianju Feng, Mingxia Wen, Tianyu Wang, Bei Huang, Mei Lin***

Institute of Citrus Research, Zhejiang Academy of Agricultural Sciences, Taizhou, China

*Correspondence:

Weiqing Zhang

[zhangweiiqngqing@126.com](mailto:zhangweiiqngqing@126.com)

Mei Lin

[hylm84712002@126.com](mailto:hylm84712002@126.com)

**Table S1. Calibration curves of the flavonoid standards.**

| Standards | Linear range  (µg/L) | Calibration curve | LOQ  (µg/kg) | *R*^2^ |
| --- | --- | --- | --- | --- |
| Tangeretin | 0.1-500 | y=44161.2x+186007.6 | 20 | 0.9965 |
| Sinensetin | 0.1-500 | y=5108.4x+14304.6 | 20 | 0.9935 |
| Nobiletin | 0.1-500 | y=12868.5x+69801.1 | 20 | 0.9902 |
| Rhoifolin | 0.1-500 | y=694.2x+1644.3 | 20 | 0.9970 |
| Vitexin | 0.1-500 | y=8181.7x+252.5 | 20 | 0.9971 |
| Diosmetin | 0.1-500 | y=25517.7x+1950.8 | 10 | 0.9987 |
| Didymin | 0.1-500 | y=297.4x+1951.6 | 20 | 0.9986 |
| Poncirin | 0.1-500 | y=319.4x+2173.2 | 20 | 0.9982 |
| Neohesperidin | 0.1-500 | y=184.7x+14823.6 | 20 | 0.9968 |
| Narirutin | 0.1-500 | y=2569.1x+108179.7 | 20 | 0.9979 |
| Naringin | 0.1-500 | y=693.5x+8612.3 | 20 | 0.9950 |
| Hesperidin | 0.1-500 | y=117.3x+10018.6 | 20 | 0.9963 |
| Hesperetin | 0.1-500 | y=3928.1x+6616.4 | 20 | 0.9980 |
| Naringenin | 0.1-500 | y=8057.5x+19827.5 | 20 | 0.9937 |
| Eriocitrin | 0.1-500 | y = 2584.2x + 12932.1 | 20 | 0.9950 |
| Neoeriocitrin | 0.1-500 | y = 420.9x +1226.3 | 20 | 0.9969 |
| rutin | 0.1-500 | y=809.3x+22674.5 | 20 | 0.9954 |

**Table S2. Calibration curves of the phenolic acid standards.**

| Standards | Linear range  (µg/L) | Calibration curve | LOQ  (µg/kg) | *R*^2^ |
| --- | --- | --- | --- | --- |
| 4-Hydroxybenzoic acid | 0.1-250 | y=21751.5x+68668.1 | 5 | 0.9971 |
| Salicylic acid | 0.1-250 | y=2208.4x+7240.3 | 5 | 0.9981 |
| Coumalic acid | 0.1-250 | y=688.3x+5399.4 | 5 | 0.9936 |
| Protocatechuic acid | 0.1-250 | y=794.6x+7727.2 | 5 | 0.9964 |
| 2,5-dihydroxybenzoic acid | 0.1-250 | y=12019.5x+24548.2 | 5 | 0.9968 |
| p-Coumaric acid | 0.1-250 | y=13918.1x+55886.3 | 5 | 0.9972 |
| Vanillic acid | 0.1-250 | y=121.2x+390.1 | 5 | 0.9977 |
| Gallic acid | 0.1-250 | y=14516.4x+23616.2 | 5 | 0.9977 |
| Caffeic acid | 0.1-250 | y=4725.8x+24545.3 | 5 | 0.9965 |
| Ferulic acid | 0.1-250 | y=1068.9x+6189.6 | 5 | 0.9968 |
| Sinapic acid | 0.1-250 | y=1172.8x+6346.5 | 5 | 0.9966 |

**Table S3. Flavonoid and phenolic acid contents in citrus peels (mg/kg DW).**

|  | Miyagawa wase | Bendizao | Ponkan | Cocktail grapefruit | Hongmeiren | Gaocheng | Yuhuanyou |
| --- | --- | --- | --- | --- | --- | --- | --- |
| Tangeretin | 224.39 ± 9.99 c | 1158.31 ± 65.52 b | 3714.90 ± 93.68 a | 216.07 ± 10.00 c | 11.45 ± 4.45 e | 136.37 ± 4.33 d | 8.71 ± 4.17 e |
| Sinensetin | 56.15 ± 2.33 c | 211.01 ± 15.55 b | 432.11 ± 49.36 a | 2.96 ± 0.51 d | 1.30 ± 0.58 d | 49.38 ± 2.91 c | nd |
| Nobiletin | 722.15 ± 22.95 d | 2964.45 ± 242.07 b | 8316.06 ± 617.74 a | 446.50 ± 36.42 d | 15.10 ± 10.00 e | 1310.66 ± 73.09 c | 1.74 ± 0.20 e |
| **ΣPMFs** | **1002.69 ± 32.69 d** | **4333.78 ± 307.33 b** | **12463.07 ± 704.03 a** | **665.53 ± 40.78 d** | **27.85 ± 14.77 e** | **1496.41 ± 77.05 c** | **10.45 ± 4.09 e** |
| Rhoifolin | 32.90 ± 1.35 cd | 4.24 ± 0.80 d | 10.61 ± 1.29 cd | 18.87 ± 2.70 cd | 238.39 ± 11.38 b | 61.18 ± 4.46 c | 1443.08 ± 126.49 a |
| Vitexin | 12.99 ± 0.42 d | 26.43 ± 1.74 c | 28.59 ± 0.82 b | 3.98 ± 0.27 f | 11.60 ± 0.42 e | 2.72 ± 0.29 g | 48.13 ± 0.99 a |
| Diosmetin | 1.69 ± 0.21 b | 1.56 ± 0.10 b | 7.76 ± 0.20 a | 0.27 ± 0.04 d | nd | 0.52 ± 0.08 c | nd |
| **ΣFlavones** | **47.59 ± 1.72 c** | **32.23 ± 2.58 c** | **46.96 ± 1.84 c** | **23.11 ± 2.78 c** | **250.00 ± 11.67 b** | **64.43 ± 4.32 c** | **1491.21 ± 126.97 a** |
| Didymin | 2205.99 ± 107.18 a | 241.12 ± 50.27 d | 700.55 ± 35.67 c | 86.68 ± 1.28 e | 2003.77 ± 110.07 b | 24.07 ± 1.74 e | nd |
| Poncirin | 1400.98 ± 91.19 a | 149.53 ± 34.45 e | 438.81 ± 23.03 c | 244.98 ± 17.38 d | 1222.24 ± 81.95 b | 65.18 ± 4.87 f | nd |
| Neohesperidin | 16682.40 ± 1205.74 a | 8568.09 ± 1265.67 d | 8976.11 ± 773.20 d | 11221.04 ± 290.94 c | 9262.47 ± 198.59 d | 15166.48 ± 894.74 b | 4.49 ± 0.48 e |
| Narirutin | 8081.18 ± 395.18 d | 265.94 ± 39.42 f | 401.36 ± 13.67 f | 11957.19 ± 174.69 b | 1761.70 ± 20.72 e | 9335.08 ± 560.47 c | 19270.36 ± 202.11 a |
| Naringin | 566.99 ± 47.58 d | 23.29 ± 2.31 d | 33.63 ± 1.68 d | 25014.93 ± 477.71 b | 132.86 ± 9.78 d | 21105.99 ± 1495.73 c | 44682.72 ± 728.66 a |
| Hesperidin | 41796.14 ± 2709.57 a | 21085.41 ± 2795.60 b | 22492.34 ± 1467.51 b | 8688.67 ± 615.43d | 22837.48 ± 805.08 b | 11102.97 ± 718.51 c | 4.45 ± 0.88 e |
| Hesperetin | 4.37 ± 0.50 a | 1.65 ±0.25 c | nd | 1.13 ± 0.15 d | 2.57 ± 0.19 b | 0.56 ± 0.06 e | nd |
| Naringenin | 4.53 ± 0.26 a | 0.25 ± 0.02 e | 0.34 ± 0.05 e | 1.75 ± 0.21b | 0.84 ± 0.12 c | 0.59 ± 0.06 d | 0.84 ± 0.04 c |
| Eriocitrin | 292.78 ± 9.81 c | 52.92 ± 1.90 d | 41.07 ± 1.58 d | 2381.78 ± 198.21 a | 53.26 ± 2.55 d | 902.13 ± 89.85 b | nd |
| Neoeriocitrin | nd | nd | nd | 3278.16 ± 88.51 b | nd | 6535.24 ± 314.20 a | 40.56 ± 1.07 c |
| **ΣFlavanones** | **71035.36 ± 4142.12 a** | **30388.21 ± 4150.44 d** | **33084.22 ± 2229.85 d** | **62876.31 ± 747.19 b** | **37277.19 ± 1093.14 c** | **64238.29 ± 3872.15 b** | **64003.41 ± 852.74 b** |
| Rutin **( Flavonol)** | **32170.54 ± 2055.94 a** | **16418.35 ± 2034.06 b** | **17249.63 ± 1242.63 b** | **10465.48 ± 532.27d** | **17623.91 ± 808.44 b** | **13635.23 ± 711.13 c** | **4.31 ± 0.88 e** |
| **ΣFlavonoids** | **104256.17 ± 5629.63 a** | **51172.56 ± 6453.32 e** | **62843.88 ± 3272.31 d** | **74030.43 ± 1073.91 c** | **55178.95 ± 1812.45 e** | **79434.36 ± 4626.59 b** | **65509.38 ± 957.95 d** |
| 4-hydroxybenzoic acid | 34.71 ± 6.62 a | 16.57 ± 1.77 d | 20.89 ± 1.51 c | 12.74 ± 0.49 e | 27.78 ± 1.31 b | 15.04 ± 0.45 de | 17.17 ± 1.02 d |
| Salicylic acid | 4.55 ± 0.29 c | 10.63 ± 0.32 a | 7.06 ± 0.60 b | 10.61 ± 0.75 a | 1.99 ± 0.07 d | 0.98 ± 0.11 e | 0.95 ± 0.07 e |
| Coumalic acid | nd | nd | nd | nd | nd | nd | nd |
| Protocatechuic acid | 9.52 ± 0.96 b | 13.57 ± 0.39 a | 4.47 ± 0.18 d | 5.20 ± 0.16 c | 4.05 ± 0.25 de | 3.64 ± 0.15 e | 1.66 ± 0.07 f |
| 2,5-dihydroxybenzoic acid | 0.10 ± 0.02 bc | 0.24 ± 0.05 a | 0.10 ± 0.03 bc | 0.10 ± 0.03 bc | 0.14 ± 0.06 b | 0.11 ± 0.02 b | 0.07 ± 0.01 c |
| p-Coumaric acid | 129.37 ± 4.28 d | 276.97 ± 9.85 b | 403.20 ± 18.11 a | 47.93 ± 1.63 f | 255.75 ± 5.72 c | 60.53 ± 1.32 e | 11.69 ± 1.06 g |
| Vanillic acid | 58.32 ± 8.10 b | 54.40 ± 6.14bc | 38.70 ± 4.03 d | 40.02 ± 2.31 d | 49.85 ± 2.59 c | 71.64 ± 4.58 a | 18.62 ± 1.23 e |
| Gallic acid | 0.75 ± 0.11 d | 0.93 ± 0.06 a | 0.80 ± 0.03 cd | 0.78 ± 0.04 d | 0.80 ± 0.03 cd | 0.85 ± 0.06 bc | 0.88 ± 0.02 ab |
| Caffeic acid | 54.97 ± 11.28 d | 284.90 ± 4.74 b | 535.05 ± 26.06 a | 46.13 ± 2.49 d | 105.82 ± 57.71 c | 7.48 ± 0.29 e | 4.47 ± 0.45 e |
| Ferulic acid | 1549.89 ± 65.41 d | 3033.13 ± 67.45 a | 2668.48 ± 123.41 b | 358.20 ± 22.57 e | 2529.17 ± 103.14 c | 366.97 ± 7.17 e | 26.81 ± 2.09 f |
| Sinapic acid | 114.44 ± 3.80 b | 241.67 ± 16.57 a | 113.69 ± 10.96 b | 71.60 ± 10.19 d | 97.71 ± 2.81 c | 47.89 ± 8.99 e | 79.28 ± 13.57 d |
| **ΣPhenolic acids** | **1956.63 ± 65.11 d** | **3933.01 ± 91.87 a** | **3792.45 ± 160.17 b** | **593.32 ± 34.85 e** | **3073.07 ± 52.76 c** | **575.13 ± 11.64 e** | **161.60 ± 17.70 f** |

The content was represented as mean ± standard deviation (n = 3). Different lowercase letters indicate significant differences between samples (*p* < 0.05). Undetected was indicated by “nd.”

**Table S4. Flavonoid and phenolic acid contents in citrus pulps (mg/kg DW).**

|  | Miyagawa wase | Bendizao | Ponkan | Cocktail grapefruit | Hongmeiren | Gaocheng | Yuhuanyou |
| --- | --- | --- | --- | --- | --- | --- | --- |
| Tangeretin | 0.63 ± 0.17 c | 1.52 ± 0.13 b | 8.92 ± 0.76 a | 1.19 ± 0.11 b | 0.35 ± 0.05 cd | 0.54 ± 0.08 c | 0.10 ± 0.02 d |
| Sinensetin | nd | nd | nd | nd | nd | nd | nd |
| Nobiletin | 2.10 ± 0.47cd | 3.85 ± 0.25 b | 27.15 ± 3.49 a | 3.76 ± 0.53 bc | 0.84 ± 0.20 d | 5.44 ± 0.41 b | nd |
| **ΣPMFs** | **2.73 ± 0.64 c** | **5.36 ± 0.35 b** | **36.07 ± 4.21 a** | **4.95 ± 0.62 b** | **1.20 ± 0.24 cd** | **5.98 ± 0.45 b** | **0.10 ± 0.02 d** |
| Rhoifolin | nd | nd | nd | nd | 31.33 ± 3.58 b | 6.72 ± 0.77 b | 699.15 ± 50.25 a |
| Vitexin | 5202.17 ± 350.18 b | 5526.16 ± 420.03 a | 4859.18 ± 356.06 c | 1330.59 ± 33.39 f | 2054.23 ± 116.47 e | 2853.66 ± 151.69 d | 5.65 ± 1.68 g |
| Diosmetin | 0.17 ± 0.12 b | 0.17 ± 0.02 b | 0.34 ± 0.03 a | 0.02 ± 0.01 d | 0.29 ± 0.04 a | 0.09 ± 0.01 c | 0.02 ± 0.00 d |
| **Σ****Flavones** | **5202.34 ± 350.10 b** | **5526.33 ± 420.02 a** | **4859.52 ± 356.06 c** | **1330.61 ± 33.39f** | **2085.84 ± 119.28 e** | **2860.47 ± 151.78 d** | **704.82 ± 48.66 g** |
| Didymin | 529.73 ± 26.89 a | 488.28 ± 37.29 b | 486.98 ± 38.86 b | 23.07 ± 2.00 d | 392.30 ± 32.63 c | 27.63 ± 2.93 d | 0.39 ± 0.14 d |
| Poncirin | 345.32 ± 20.95 a | 291.92 ± 28.86 b | 323.66 ± 18.78 a | 59.39 ± 4.49d | 256.86 ± 18.60 c | 68.90 ± 15.75 d | nd |
| Neohesperidin | 2279.80 ± 142.12 b | 2614.42 ± 264.82 b | 2291.54 ± 195.32 b | 2481.40 ± 81.61b | 978.33 ± 146.69 c | 7079.99 ± 651.90 a | 2.82 ± 0.66 d |
| Narirutin | 2040.60 ± 70.64 d | 488.32 ± 15.05 e | 392.58 ± 30.16 e | 3444.09 ± 122.72c | 506.69 ± 21.88 e | 4011.40 ± 465.78 b | 5788.75 ± 226.88 a |
| Naringin | 159.99 ± 18.52 d | 44.12 ± 2.23 d | 26.36 ± 2.07 d | 6553.24 ± 213.99c | 33.22 ± 1.30 d | 8765.71 ± 1029.73 b | 13705.90 ± 338.57 a |
| Hesperidin | 5459.21 ± 466.92 bc | 6276.84 ± 695.28 a | 5536.16 ± 482.35 b | 1760.21 ± 182.92 e | 2365.43 ± 430.08 d | 5014.56 ± 352.85 c | 4.77 ± 1.68 f |
| Hesperetin | 3.57 ± 0.17 c | 26.23 ± 1.82 a | 5.28 ± 0.43 b | 0.99 ± 0.04 d | 1.39 ± 0.05 d | 4.64 ± 0.29 b | nd |
| Naringenin | 4.70 ± 0.26 a | 4.31 ± 0.12 b | 1.18 ± 0.05 e | 3.45 ± 0.22 c | 0.79 ± 0.04 f | 1.54 ± 0.04 d | 0.75 ± 0.07 f |
| Eriocitrin | 77.28 ± 2.08 c | 32.12 ± 1.27 de | 33.48 ± 2.81 d | 921.21 ± 18.56 a | 15.58 ± 1.02 e | 344.08 ± 29.51 b | nd |
| Neoeriocitrin | nd | 1.45 ± 0.23 c | nd | 841.25 ± 30.72 b | nd | 1771.87 ± 220.46 a | 74.34 ± 3.21 c |
| **ΣFlavanones** | **10900.20 ± 712.70 d** | **10268.00 ± 1020.91 de** | **9097.22 ± 754.01 e** | **16088.93 ± 464.43 c** | **4550.59 ± 625.07 f** | **27090.32 ± 2707.39 a** | **19577.73 ± 552.3 b** |
| Rutin **( Flavonol)** | **4281.78 ± 369.84 c** | **5004.16 ± 554.89 b** | **4403.82 ± 363.63 c** | **2254.01 ± 100.82 d** | **1823.54 ± 267.28 e** | **6343.83 ± 487.54 a** | **4.31 ± 1.47 f** |
| **Σ****Flavonoids** | **20387.04 ± 1412.78 bc** | **20803.86 ± 1969.61 b** | **18396.62 ± 1452.90 c** | **19678.50 ± 592.12 bc** | **8461.17 ± 1005.54 d** | **36300.60 ± 3334.15 a** | **20286.95 ± 584.68 bc** |
| 4-hydroxybenzoic acid | 7.64 ± 0.58 b | 3.15 ± 0.24 d | 5.32 ± 0.25 c | 5.22 ± 0.39 c | 7.09 ± 0.26 b | 7.90 ± 0.23 b | 14.18 ± 2.15 a |
| Salicylic acid | 2.11 ± 0.13 de | 2.08 ± 0.33 de | 2.84 ± 0.15 c | 4.16 ± 0.18 b | 2.24 ± 0.28 d | 6.07 ± 0.11 a | 1.98 ± 0.16 e |
| Coumalic acid | nd | nd | nd | nd | nd | nd | nd |
| Protocatechuic acid | 5.90 ± 1.08 b | 3.38 ± 0.21 d | 3.51 ± 0.11 d | 7.66 ± 0.60 a | 5.01 ± 0.12 c | 6.93 ± 1.50 a | 4.09 ± 0.27 d |
| 2,5-dihydroxybenzoic acid | 0.59 ± 0.29 b | 0.19 ± 0.01 d | 0.21 ± 0.01 d | 0.24 ± 0.02 cd | 1.12 ± 0.06 a | 0.36 ± 0.02 c | 0.17 ± 0.03 d |
| p-Coumaric acid | 1.10 ± 0.11 d | 1.86 ± 0.03 b | 1.35 ± 0.03 c | 0.49 ± 0.02 f | 2.42 ± 0.04 a | 0.84 ± 0.02 e | 2.40 ± 0.15 a |
| Vanillic acid | 26.58 ± 2.03 c | 20.34 ± 1.21 d | 20.25 ± 1.27 d | 34.23 ± 2.04 b | 26.00 ± 2.04 c | 40.40 ± 2.91 a | 40.25 ± 2.31 a |
| Gallic acid | 0.69 ± 0.03 b | 0.49 ± 0.02 d | 0.58 ± 0.03 c | 0.74 ± 0.09 ab | 0.78 ± 0.02 a | 0.78 ± 0.05 a | 0.52 ± 0.03 d |
| Caffeic acid | 16.69 ± 0.61 c | 18.69 ± 2.37 c | 35.61 ± 1.57 b | 45.79 ± 1.78 a | 34.82 ± 1.02 b | 13.09 ± 0.45 d | 44.73 ± 3.42 a |
| Ferulic acid | 345.05 ± 37.69 d | 507.17 ± 26.97 b | 539.54 ± 8.03 a | 169.03 ± 2.52 e | 452.82 ± 22.95 c | 150.84 ± 2.39 e | 52.35 ± 4.33 f |
| Sinapic acid | 16.96 ± 3.07 e | 46.85 ± 3.70 b | 38.03 ± 1.03 c | 43.18 ± 5.19 b | 27.05 ± 1.74 d | 20.49 ± 0.43 e | 55.07 ± 8.17 a |
| **Σ****Phenolic acids** | **423.32 ± 44.04 d** | **604.20 ± 21.90 b** | **647.23 ± 10.00 a** | **310.73 ± 7.62 e** | **559.35 ± 24.34 c** | **247.71 ± 3.45 f** | **215.74 ± 20.53 g** |

The content was represented as mean ± standard deviation (n = 3). Different lowercase letters indicate significant differences between samples (*p* < 0.05). Undetected was indicated by “nd.”

**Table S5.** **Correlations among phenolic compounds in citrus cultivars**


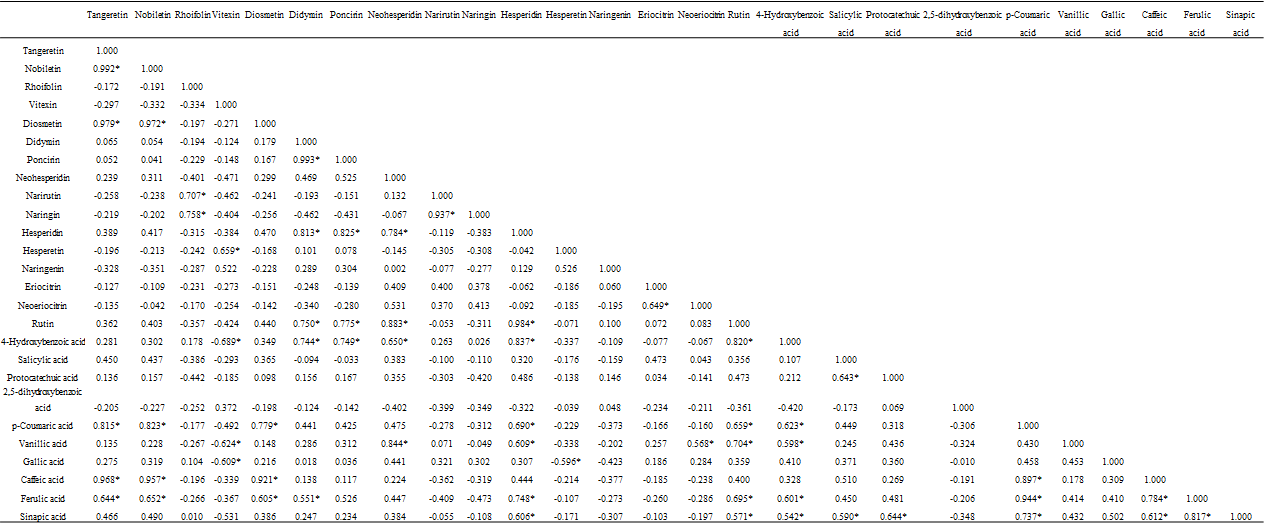

Supplement: Supplementary file 1 [file Table_1.DOCX]
